# Focal-Plane Change Triggered Video Compression for Low-Power Vision Sensor Systems

**DOI:** 10.1371/journal.pone.0006384

**Published:** 2009-07-24

**Authors:** Yu M. Chi, Ralph Etienne-Cummings, Gert Cauwenberghs

**Affiliations:** 1 Department of Electrical and Computer Engineering, University of California San Diego, La Jolla, California, United States of America; 2 Neurobiology Section, Division of Biological Sciences, University of California San Diego, La Jolla, California, United States of America; 3 Department of Bioengineering, Jacobs School of Engineering, University of California San Diego, La Jolla, California, United States of America; University of Southern California, United States of America

## Abstract

Video sensors with embedded compression offer significant energy savings in transmission but incur energy losses in the complexity of the encoder. Energy efficient video compression architectures for CMOS image sensors with focal-plane change detection are presented and analyzed. The compression architectures use pixel-level computational circuits to minimize energy usage by selectively processing only pixels which generate significant temporal intensity changes. Using the temporal intensity change detection to gate the operation of a differential DCT based encoder achieves nearly identical image quality to traditional systems (4dB decrease in PSNR) while reducing the amount of data that is processed by 67% and reducing overall power consumption reduction of 51%. These typical energy savings, resulting from the sparsity of motion activity in the visual scene, demonstrate the utility of focal-plane change triggered compression to surveillance vision systems.

## Introduction

Video compression is among the most computationally intensive tasks in current imaging technology [1] [2]. Advanced compression schemes like H.264 provide, simultaneously, high compression rates and low visual distortion. Implementation, however, is costly in both terms of power consumption and hardware complexity and is ill-suited for mobile applications. For situations requiring a low power, long term wireless vision sensor, an alternative approach is justified for two reasons. First in many sensor applications, like surveillance, scenes are predominantly static - necessitating a sensor platform that does not expend energy processing irrelevant data. Secondly, the tradeoffs between bandwidth, power and visual quality are different. The first two must be prioritized with the provision of maintaining an image sufficient to identify events and subjects of interest.

Although the use of differential coding and motion compensation minimizes the data output rate for periods in activity for conventional video encoders, the entire encoding chain including sensor, ADC and DSP must operate continuously [2]. For static, unchanging scenes, such systems inefficiently dissipate energy processing pixels that do not convey any new or meaningful information. Thus, for long term surveillance applications, it is the power usage by the sensor and DSP in monitoring the scene that will dominate the operating lifetime of the system. For a truly low power system, this energy waste must be minimized.

Our approach utilizes the possibilities afforded by focal-plane processing in CMOS image sensors [3]–[12]. Many previous research efforts have successfully demonstrated the viability of implementing focal-plane spatial transforms [5]–[9] to facilitate highly power efficient image compression. Although such solutions are ideal for single snapshots, they do not account for the temporal redundancy in full motion visual information, and are not ideal for video rate applications. In this paper, we present compression architectures that employ focal-plane change detection as a temporal processor, rather than spatial, to selectively encoded video data to reduce the power consumption in scenarios where static scenes dominate.

**Figure 1:**
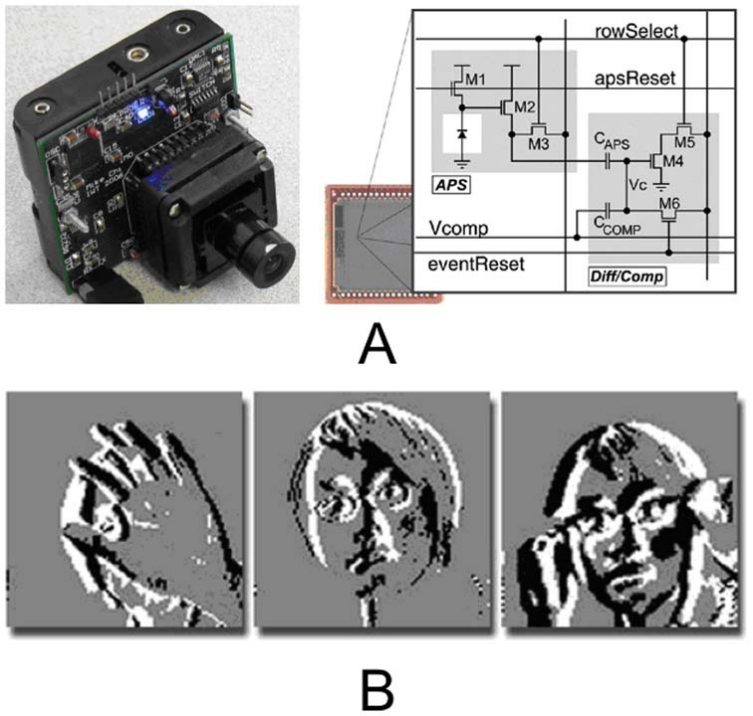
(A) Motion-based imaging integrated surveillance system with CMOS image sensor that performs change detection at the pixel level. (B) Sample change detection output of the CMOS image sensor. The system operates on 4 AA batteries, and includes a 16-bit microcontroller for integrated video compression and power management.

Figure 1a shows the circuits of a CMOS image sensor with focal-plane change detection [11]. The imager detects, in each pixel, changes in intensity exceeding a positive and negative threshold, and codes pixel locations of these change events, along with the intensity for any pixel on demand. This CMOS imager forms the basis for energy efficient video compression schemes in this paper that uses the gating of change events to save on the cost of data conversion and computation in the encoding of frame fragments that have insignificant change.

A system integrating the CMOS image sensor [13], [14] with external supporting circuitry and microprocessor is shown in Figure 1b. The image sensor provides the temporal pixel intensity change trigger as well as analog video signals, which are digitized by an external ADC on demand. Image processing and compression operations are then undertaken by the microcontroller produce a compressed digital output for connection with a wireless communications system.

The basic operation and simple compression architecture was described in [11], [13], [14]. In this paper we expand on the compression architecture by adding an entropy encoder, along with differential encoding to further reduce the data rate. In addition, the architecture has been generalized and compared with other related change triggered encoding schemes. Finally, the distortion and power efficiency of the system is analyzed, and the performance characterized on benchmark surveillance video data.

### 1. Compression Architectures Overview

**Figure 2:**
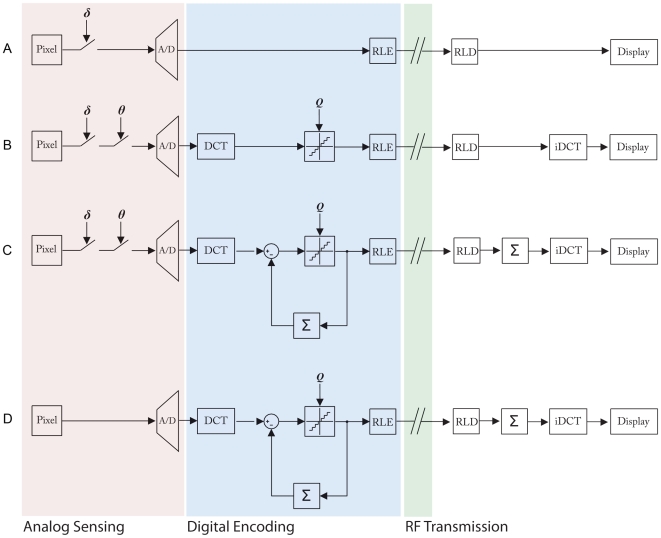
Block Diagram of the four encoders analyzed. (A) Change Triggered Pixel Refresh, (B) Change Triggered DCT Refresh, (C) Change Triggered DCT DPCM and the conventional (D) DCT DPCM encoder [16].

The overall design goals for the compression architectures are targeted for operation on a power constrained platform with limited processing capabilities over a low bandwidth wireless network. Figure 2 shows the block diagram for each of the encoders with the signal chain starting at the pixel and ending at the decoded image at the receiver with each of the operations.

### 2. Change-Triggered Pixel Refresh (CT Pixel Refresh)

The simplest form of video compression involves sending only pixels that exceed a set intensity change threshold [15] from frame to frame after an initial keyframe (Fig. 2a). An analog threshold value, δ, sets the trigger point for the intensity change detection circuit. Pixels which have an intensity change greater than the magnitude of δ are flagged as significant, digitized by the ADC and transmitted.

One immediate limitation of this coding method is the cost of transmitting pixel locations in addition to the actual updated intensity value. While conventional raster scanned image readouts implicitly embed pixel addresses in the output order, selectively sending pixels that change require an address tag for each pixel. Simply attaching the address for a pixel is unfeasible since even for a small 128×128 imager, each address tag would require 14-bits, almost twice the amount of the pixel data. In most cases, this would effectively negate any compression gain, and can very well lead to an expansion in the output data rate.

However, under the assumption that the majority of pixels from frame to frame do not change, and the assumption that pixels which do change are adjacent to each other, run-length encoding (RLE) can be used as a simple and efficient method to encode pixel positions. Here the array of pixels is treated as a 1-D vector (through a raster scan order). The data stream begins by transmitting the run length (number) of unchanged pixels before the first changed pixels. When the first changed pixel is encountered, a second run-length is computed for the number of contiguous pixels from that location that do change. Finally, the actual intensity values of these changed pixels are appended to the bit stream. Both run lengths and pixel values are coded as 8-bit values.

All of the image processing and decision making is performed inside the pixel array. Although the performance of this coding method suffers compared to the more advance DCT block based approaches, the advantage lies in the sheer simplicity of implementation. A full digital processor (microcontroller or DSP) is not needed, only a simple counter for the run-lengths followed by some basic logic for interfacing with a transmitter.

### 3. Change Triggered DCT Refresh (CT DCT Refresh)

The main shortcoming of the pixel refresh coding is that it does not adequately exploit the large spatial redundancy inherent in image data to further increase the compression rate. Transform coding is widely used in image and video compression to more efficiently represent image data in the frequency domain. The discrete cosine transform (DCT) is a near optimal transform for natural images for compacting the image input into a few spectral coefficients.

Figure 2b shows the block diagram for the CT DCT Block Refresh system. Encoding begins by treating each pixel as a member of a block rather than an independent entity in the pixel refresh case. The pixel array is partitioned into 8 by 8 pixel blocks. A new parameter, Θ, is used to set the block change threshold. If a block contains Θ number of pixels that exceed the δ change threshold, then it is flagged as significant for coding. These two parameters are used selectively gate which blocks to process. Blocks deemed inactive are wholly ignored in the subsequent signal chain.

Significant blocks are digitized followed with the DCT to produce a matrix of 8 by 8 DCT coefficients. A uniform quantization factor, *Q*, is used to scale and truncate the transformed image. Higher values of *Q* result a heavier quantization, which sets more of the DCT coefficients to zero.

Coefficients are vectorized in the standard zigzag fashion which ranks coefficients in order of increasing frequency. Each non-zero coefficient has a 4-bit value indicating the number of preceding zeros followed by the actual value. Since the DCT transform outputs 12-bit values from the 8-bit pixel data, the actual number of bits to required to represent a coefficient is simply, 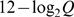, for the value plus the 4-bit run length.

The DCT coding architecture generally performs much better than single pixel coding, since it compacts a whole block of pixels into a relatively small number of coefficients and is scalable by setting *Q*. It is important to note that this and the pixel update refresh scheme require no frame buffer, just as in the CT Pixel Refresh case. For the DCT Block Refresh, only the memory required to code one 8×8 block of pixels is needed.

### 4. Change Triggered DCT Differential Pulse Code Modulation (CT DCT DPCM)

More compression gain can be realized by sending not just the transforms of the raw block data, but by transmitting the difference of the transforms using differential pulse code modulation (DPCM), although at a cost of now requiring a frame buffer. Typically values inside a block exhibit a large correlation from frame to frame, even as they undergo change. Sending a differentially coded update, rather than the raw value takes advantage of this correlation to reduce the amount of data that is needed to be transmitted.

The coding scheme operates as follows. An incoming block is flagged as significant and DCT coded if Θ number of pixels exceed the δ threshold, in the exact same manner as before. However, instead of quantization as before, the transformed coefficients are subtracted with the previous frames coefficients to produce a differential value. It is this value that is quantized by *Q* and run length coded (Fig. 2c).

In addition to sending the differential coefficient update, the encoder also takes the quantized differential coefficients and uses it update the frame buffer so that the encoder always has the same compressed (distorted) copy of the previous frame's image. This enables the encoder to operate as a closed look DPCM, which eliminates the accumulation of error arising from quantization and small drifts over time.

The differential encoder will usually produce a significantly reduced data rate for the same visual quality (*Q*) since it de-correlates the pixel data in both space and time, resulting in more zeros in the DCT coefficient matrix. However, it comes at a cost of a frame buffer, which may or may not be a significant penalty. For low resolution imaging, the frame buffer could very well fit in the internal memory provided by the digital processor.

### 5. Conventional Closed Loop DCT Differential Pulse Code Modulation (DCT DPCM)

As a reference, a conventional DCT DPCM encoder [16] was implemented to provide a benchmark against the other three coding architectures. The encoder operates in the same fashion as the CT DCT DPCM, except without the change triggered block processing. Each block is continuously processed, irrespective of the change detection circuitry. This serves as a baseline to show the power and bandwidth savings of the CT encoders while assessing the impact on distortion and image quality.

## Discussion

The power consumption of the video encoding system can be divided into three parts - the energy consumed by the pixel array and ADC to acquire the image, the energy consumed by the digital processor to process and compress the data, and finally the energy required to transmit the resulting bit stream, 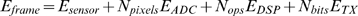



Conventional video coding systems are very successful in reducing the power required at the transmission channel by compressing the image data, hence reducing the total number of bits that are sent. While all modern coding architectures transmit a minimal of data during periods of low activity, the sensor and digital processor must still be operated continuously at full power in order to make that determination, even if the scene does not vary. Therefore, in scenarios where only intermittent visual activity is observed, the static power dissipation of the sensor, ADC and processor will limit the lifetime of the sensor.

The pixel-level change detection framework addresses the power consumption problem by enabling the system to not only efficiently control the power consumption of the transmitter, but also the ADC and digital processor by detecting activity at the focal-plane. Here all operations are gated by the presence of intensity changes (motion). This reduces the static power dissipation of the system to only the amount required to operate the pixel array, which is typically orders of magnitude less than the external processor.

For the simple pixel refresh encoder power consumption is directly related to the number of pixels that change. The energy required to code one frame is the static energy consumed by the sensor array, the energy required to digitize changed pixels and the energy required the transmit the zero run-length and pixel values 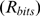.
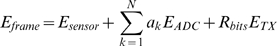



Here 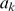 represents a gating variable for each pixel indicating whether or not it has crossed the change threshold. The energy consumed by the digital processing for this architecture can be considered negligible since only a simple counter is required to tabulate the zero change run lengths.

Switching over to the CT DCT based encoders adds an additional factor that accounts for the energy used to perform the DCT on a block. 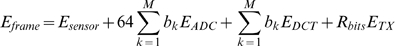



The variable 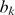 gates the M 8×8 blocks to only perform the ADC and DCT 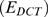 operations over blocks that exhibit change.

For the baseline DPCM encoder, power consumption is simply the constant cost of operating the sensor, ADC and performing the DCT on each block plus the energy cost of transmitting the output bit stream.
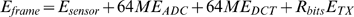



It should be observed that the two last encoding methods also require a frame buffer, but depending on image size and memory type (on-die SRAM in the processor), may only incur a negligible increase in amount of power dissipation.

### Implementation Example

Actual power consumption figures are heavily dependent on hardware implementation. However, an approximate model for the power efficiencies of each architecture can be modeled using known figures from available components. For this paper, the hardware used in [13] was used as a model for the sensor and digital signal processing energy costs.

Table 1 shows the energy cost of each operation. The energy consumed by the sensor was derived from [11] which shows that the change detection sensor requires 4 mW to power a 90×90 pixel array at 30 frames/second, corresponding to an energy expenditure of 16.5 nJ/pixel. The ADC used was a 1MSPS model with a power consumption figure of 3.9 mW. Since each pixel is one sample, the energy to digitize a pixel is 3.9 nJ.

**Table 1:** Summary of Power Usage.

Architecture	Sensor/ADC^1^	Image Processing	Transmission
DCT DPCM^1^	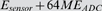	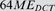	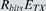
CT DCT DPCM	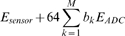	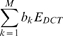	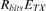
CT DCT Refresh	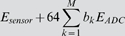	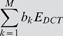	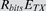
CT Pixel Refresh	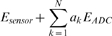	0	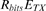

1 In the DCT DPCM encoder, 64*ME_ADC_* is equivalent to the CT DCT ADC energy cost where all the *b_k_* gating variables are equal to 1. Likewise for the 64*ME_DCT_* component.

For the power consumption of the digital processor, the PIC32 on the board requires 77.6 mW at the operating frequency of 20 MHz. A fast, integer only DCT implementation [17] can be performed with 2450 operations. This amounts to 9.5 µJ to transform one 8×8 block.

The transmitter was based on a commonly available, low power 2.4 GHz ZigBee modules. From the parameters on the datasheet, the equivalent energy to transmit a single bit of data is 224 nJ.

It is worth noting that different figures can be easily obtained by varying the implementation. For example an improved sensor or custom logic for the DCT would significantly reduce the power consumption at each of those stages. However, the figures here serve to provide not only an estimate of real world power efficiency, but to also illustrate the tradeoffs and gains in using the focal-plane CT circuits to manage the power usage of the video encoder. [Table pone-0006384-t002]

**Table 2 pone-0006384-t002:** Components and References for Power Estimation Figures.

Component	Power Consumption	Reference
Change Detection Imager	4 mW (16.4 nJ/pixel)	[11]
ADC	3.9 mW (3.9 nJ/pixel)	TI ADS7886 1MSPS SAR ADC
Processor	77.6 mW (3.9 nJ/op)	Microchip PIC32, [13]
Transmitter	57.4 mW (224 nJ/bit)	TI CC2420 2.4GHz Transceiver

## Methods

To fully evaluate the performance of the focal-plane change detection based video coding architectures, computer models were used to simulate the operation of the sensor and video coder. This allowed the architectures to operate on a fixed data set consisting of well-known video test sequences as well as to fully characterize the effects on distortion, compression rate and power consumption of each encoding parameter (δ, Θ and *Q*).

The test sequence *hall.cif* is a commonly used surveillance type reference video to evaluate video coding architectures. The 200 frame CIF (352×288) resolution file contains imagery similar to raw video output of the sensor in Figure 1. The compressed outputs of this sequence were used to generate the data for each of the compression architectures.

A rate-distortion (RD) plot is a useful tool to compare the performance of each encoding architecture by plotting the distortion introduced by the encoder versus the compression rate. Distortion is expressed using the peak signal-to-noise ratio (PSNR) of the maximum pixel value versus the mean-square-error (MSE) of the encoded video data from the original source. 
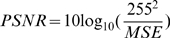



Compression rate is normalized to bits per pixel which is obtained by taking the size of the compressed bit stream divided the total number of pixels encoded. Hence a rate of 1 bit/pixel corresponds to a compression ratio of 8∶1, since the original pixel data contains 8 bits. Lower numbers indicated a higher compression ratio and smaller output data rate.

## Results

**Figure 3:**
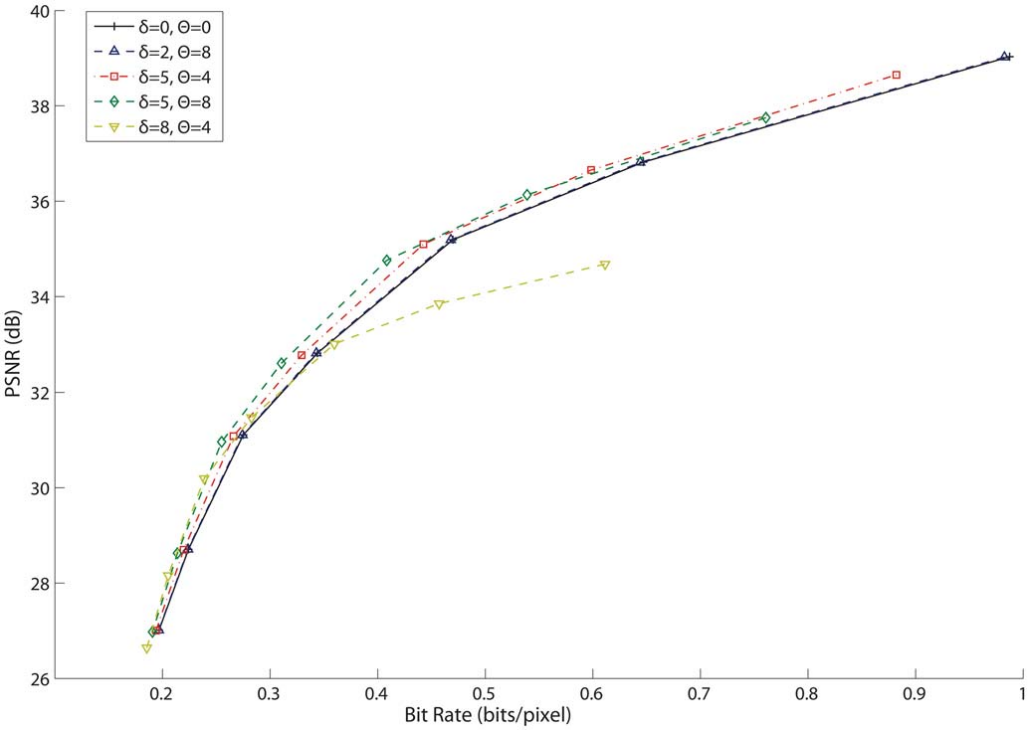
Rate-Distortion curves by varying δ and Θ for the CT DCT DPCM encoder with *Q* ranging from {16, 24, 32, 48, 64, 96, 128}. The δ = 0 and Θ = 0 case corresponds to the non change triggered baseline (DCT DPCM). The curve where δ = 5 and Θ = 8 offers the best trade-off in terms of the compression performance and introduced error.

Before a full comparison of each architecture could be conducted, it was necessary to determine the optimal parameters for δ and Θ for the CT DCT encoders. Figure 3 shows the rate-distortion curve for both the baseline DCT DPCM encoder and the CT DCT DPCM encoders. Each of the points was generated by varying the parameter, *Q* from {16, 24, 32, 48, 64, 96, 128} to set the distortion level and output data rate.

Performance of the encoder matches closely to the non-change triggered encoder except for the case where δ = 5, Θ = 8 curve when the distortion rapidly increases due to insensitivity to actual change events. This sets the optimal threshold that will reject low-level noise but not actual events of interest.

Interestingly, in all of the cases where the CT is active, the CT DPCM encoder actually performs better from a rate-distortion standpoint – the gain in bandwidth reduction due to the CT is greater than the increase in distortion due to discarding inactive blocks.

**Figure 4:**
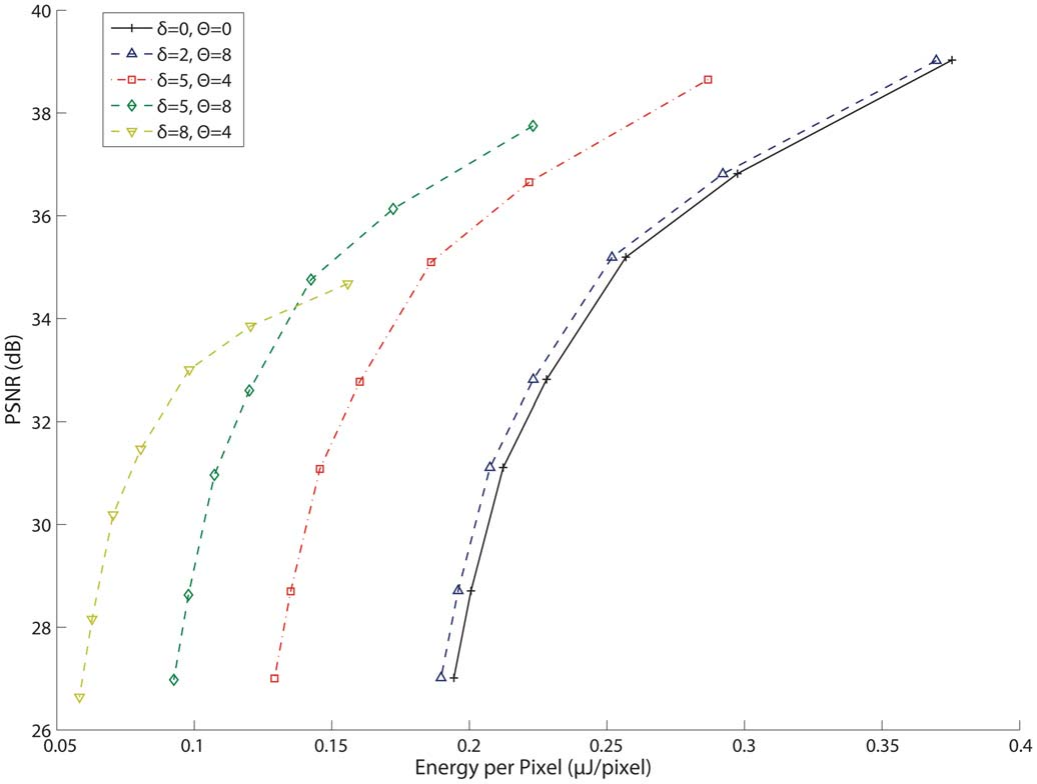
Energy-Distortion curves by varying δ and Θ for the CT DCT DPCM encoder under the same conditions as Fig. 3. Again, the δ = 5 and Θ = 8 case is optimal in using the least amount of energy per pixel while maintaining distortion levels similar to the conventional DCT DPCM encoder. Increasing the thresholds to δ = 8 and Θ = 4 for further energy savings introduces distortions significantly limiting the achievable PSNR.

Figure 4 shows a second related graph that compares the energy expenditure versus distortion (ED), where the energy per pixel was calculated using the numbers described in the previous section. As expected, for low values of δ and Θ, the energy and distortion curves match closely to the conventional DCT DPCM encoder, since simple noise was enough to trigger the encoding of a block resulting in very few rejected blocks. Increasing the thresholds shifts the curve leftward, corresponding to a reduction in energy expenditure, while largely maintaining constant PSNR values until the (8,4) case.

For the previously found optimal values of δ = 5 and Θ = 8, the reduction in the amount of blocks processed was 67%, leading to an overall 51% reduction in power consumption at a minimal impact in PSNR. Increasing the thresholds beyond this point further reduces the power consumption, but distorts the ED graph, indicating data loss.

This case illustrates the advantages of going from simple conventional DCT DPCM to using a CT DCT DPCM encoder. Significant power savings can be achieved by incorporating the power management as upstream in the signal chain as possible. For this sequence with moving events, the reduction in power was roughly one half by using the CT to gate the processing of blocks.

### Comparison of Each Encoder

**Figure 5:**
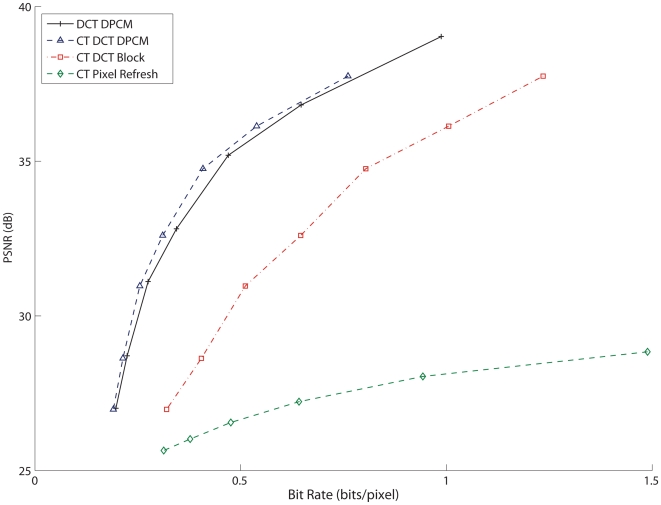
Rate-Distortion curves for each encoding algorithm. The DCT encoders were set to δ = 5 and Θ = 8, with Q ranging from 16 to 128, and δ for the CT Pixel Refresh encoder ranged from 2 to 15. As shown previously, the CT DCT DPCM encoder has the best rate-distortion.

The next step was to evaluate the performance of each architecture type. Rate-distortion curves (Figure 5) were generated by varying the *Q*, used to quantize the DCT coefficients and by varying δ for the simple CT Pixel Refresh encoder. As expected, the DCT based compression architectures all performed similarly in terms of distortion, which is largely dependent on the quantization factor *Q*. However, the DCT Refresh architecture trails the two DPCM architectures in compression ratio at an equivalent PSNR since more data is needed to transmit an entire block rather than just a differential update. The CT Pixel Refresh encoder is generally suboptimal and cannot achieve low distortion levels even at a high bit rate, but it is important to keep in mind the simplicity of implementation. In general the CT DCT DPCM encoder has the best rate-distortion tradeoff, but with a slightly higher overall distortion level than the conventional DCT DPCM encoder.

**Figure 6:**
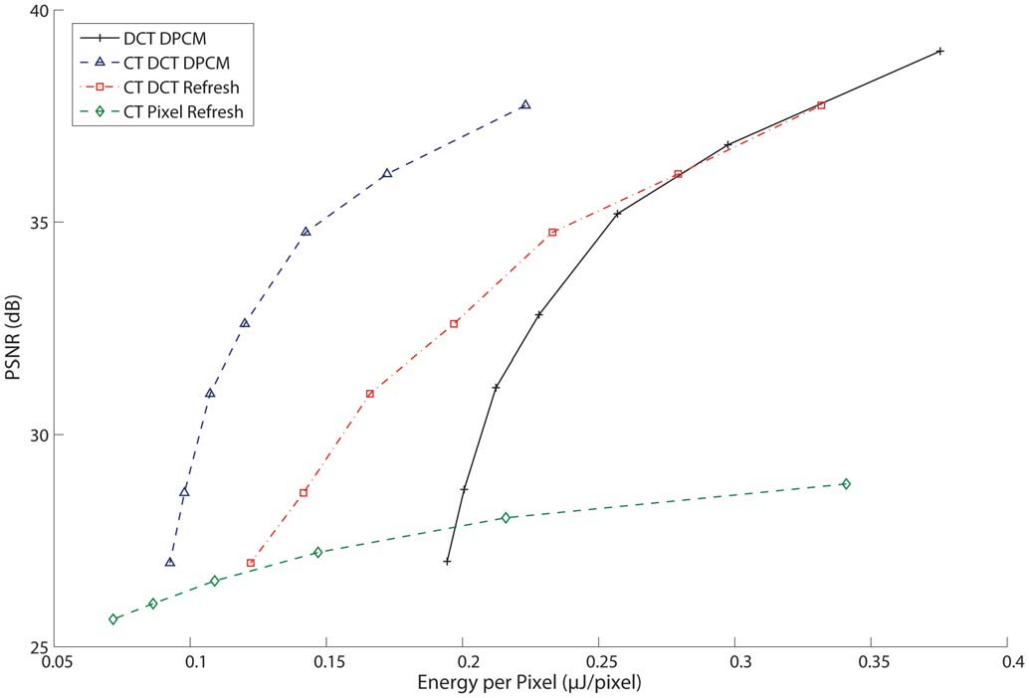
Energy-Distortion curves for each encoding algorithm, under same conditions as in Figure 5. The CT DCT DPCM encoder has the greatest efficiency in achieving a distortion level with the least amount of energy required to process and transmit the video.

Similarly, an ED graph (Figure 6) was generated for each separate architecture in the same manner. The ED graph shows that the CT DCT DPCM encoder has the best visual quality to energy expenditure ratio - achieving the low distortion levels of the DCT DPCM encoder while minimizing the energy usage by discarding irrelevant blocks. The CT DCT Block coder has good performance at high levels of *Q*, but is hampered by the energy needed to transmit more data at less aggressive DCT quantization levels due to the inefficiency in sending full block refreshes rather than differential updates. Again, the CT Pixel Refresh encoder is generally suboptimal because of the high data rates generated by sending pixel updates rather than the more efficient method of sending DCT block coefficients, resulting in increased energy used at the transmitter. It is worth noting, however, that at very high levels of distortion, the CT Pixel Refresh coder expends very little energy, albeit with a significantly impaired visual quality.

**Figure 7:**
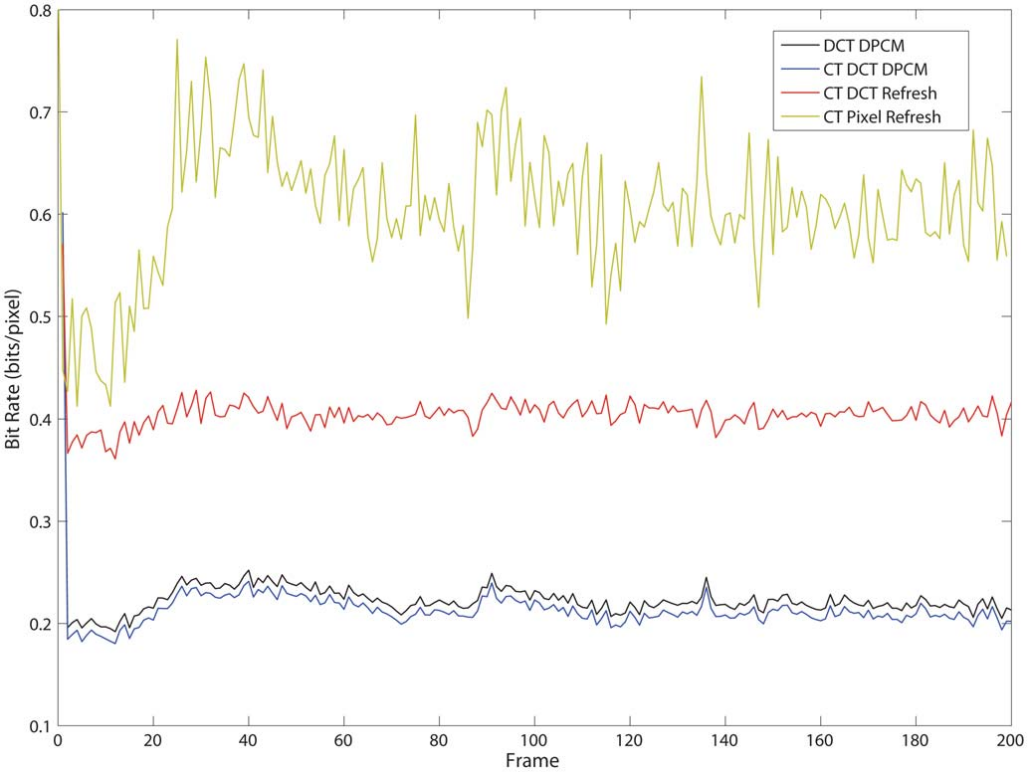
A chart of the compression rate for each frame in the *hall.cif* test sequence. The parameters used were δ = 5, Θ = 8 and *Q* = 96 for the DCT based encoders and δ = 7 for the CT Pixel Refresh encoder. The CT Pixel Refresh encoder is least optimal, and incurs a large initial transmission cost of 8 bits per pixel (keyframe, no compression).

Figure 7 shows the dynamic compression performance of the encoder over time at the point Q = 96, δ = 5 and Θ = 8 for the DCT encoders and δ = 7 for the CT Pixel Refresh. The compression rate in bits/pixel for a single frame is plotted for all 200 frames in the sequence. This illustrates that the DCT based encoders generally have much better control over the data rate (through setting *Q*) than the pixel refresh encoder, which is much more sensitive to both noise and actual observed change. In addition, the DCT DPCM encoders are about twice as efficient as the DCT refresh encoder. Note that the CT Pixel Refresh encoder requires a full uncompressed encoding of the initial frames at 8bits/pixel, incurring an initial coding and transmission cost significantly higher than the block based methods.

**Figure 8:**
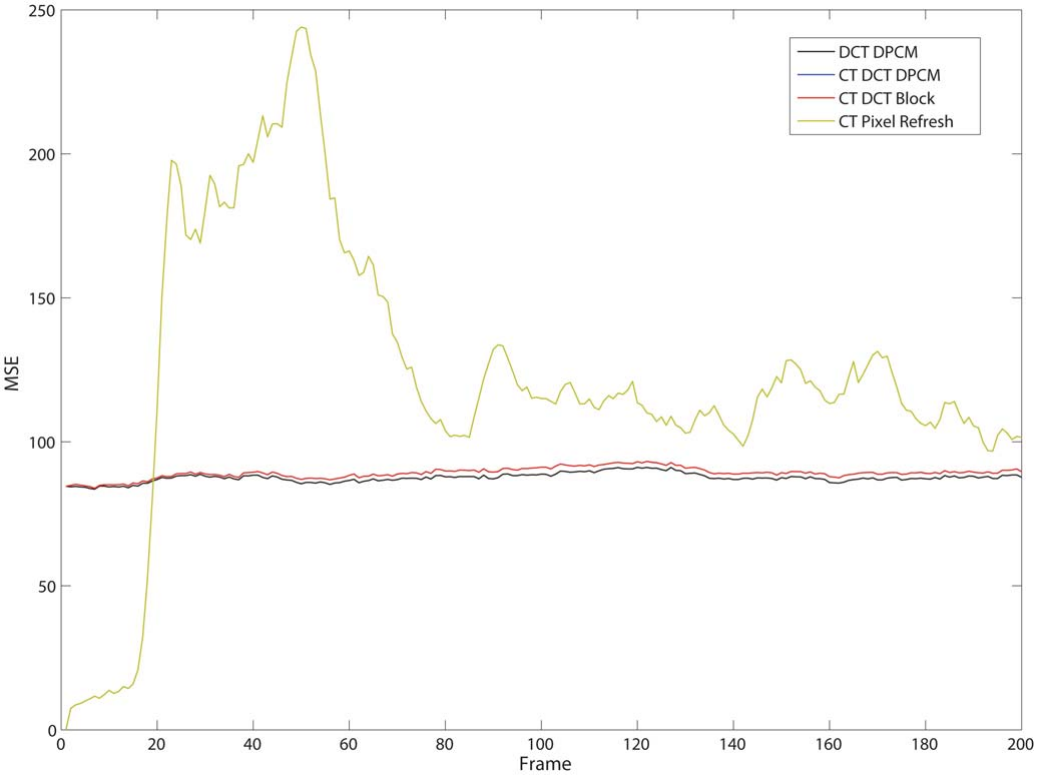
A chart of the distortion level (MSE) for each frame in the *hall.cif* test sequence. The parameters used were δ = 5, Θ = 8 and *Q* = 96 for the DCT based encoders and δ = 7 for the CT Pixel Refresh encoder as in Figure 7. The two CT DCT encoders have nearly identical distortion levels to each other and do not appear as distinct lines.

Next, the dynamic distortion rate of the encoder output is shown by plotting the MSE of a single frame over the entire sequence (Figure 8). As expected, due to the single pixel nature of the CT Pixel Refresh, the distortion varies widely and is heavily dependent on scene content. In contrast, all of the DCT encoders were able to maintain a nearly constant and similar distortion level (set by *Q*). Significantly, despite the lack of feedback inherent in the design of the CT mechanism at the focal-plane, the drift between the reference fully closed loop DPCM encoder and the CT encoders was minimal suggesting that aggregating the change detection over a block of pixels simultaneously minimized the effect of noise (a few pixels reporting change) while preserving sensitivity to actual change events (many pixels reporting change).

**Figure 9:**
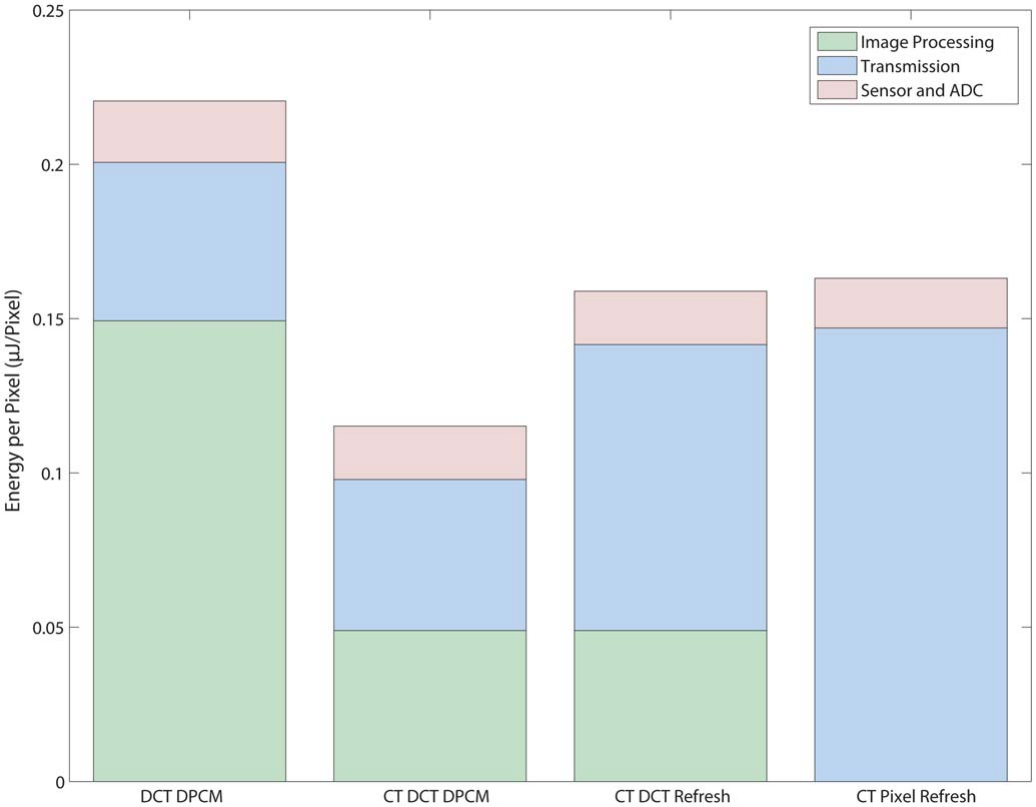
Energy allocation for each encoder using the same operating parameters as Figures 7 and 8. The use of the CT significantly decreases the amount of energy necessary for image processing since only a fraction of the blocks are transformed. Sensor and ADC energy costs are nearly equal, with the constant sensor energy usage as main factor. Although the CT Pixel Refresh Coder requires a minimal of computation cost, this is offset by the decrease in compression efficiency and higher energy usage at the transmitter.

A breakdown of the power usage by component is shown in Figure 9 at the same operating points as Figure 7 and 8 and shows the tradeoffs in using each encoding scheme. Power consumption of the imager and ADC is largely dominated by the sensor in all cases and is essential constant and smaller than the other two components in all cases. DPCM encoding incurred the least energy cost in transmission due to the compression efficiency of sending differential DCT coefficient updates. However, the baseline DCT DPCM encoder has large processing cost, since each block in every frame was continuously processed. Moving to the CT Block Refresh saved on processing energy, since only significant blocks were transformed, but at a cost of decreased compression efficiency and increased bandwidth and transmitter energy. The CT DCT DPCM encoder, on the other hand, can be viewed as the optimal solution since it had both the compression efficiency of the baseline as well as the processing efficiency as a result of using the CT to selectively code blocks. In the case of the pixel refresh, while the digital signal processing cost was minimal, the cost of transmission was significant, because of compression inefficiencies.

**Figure 10:**
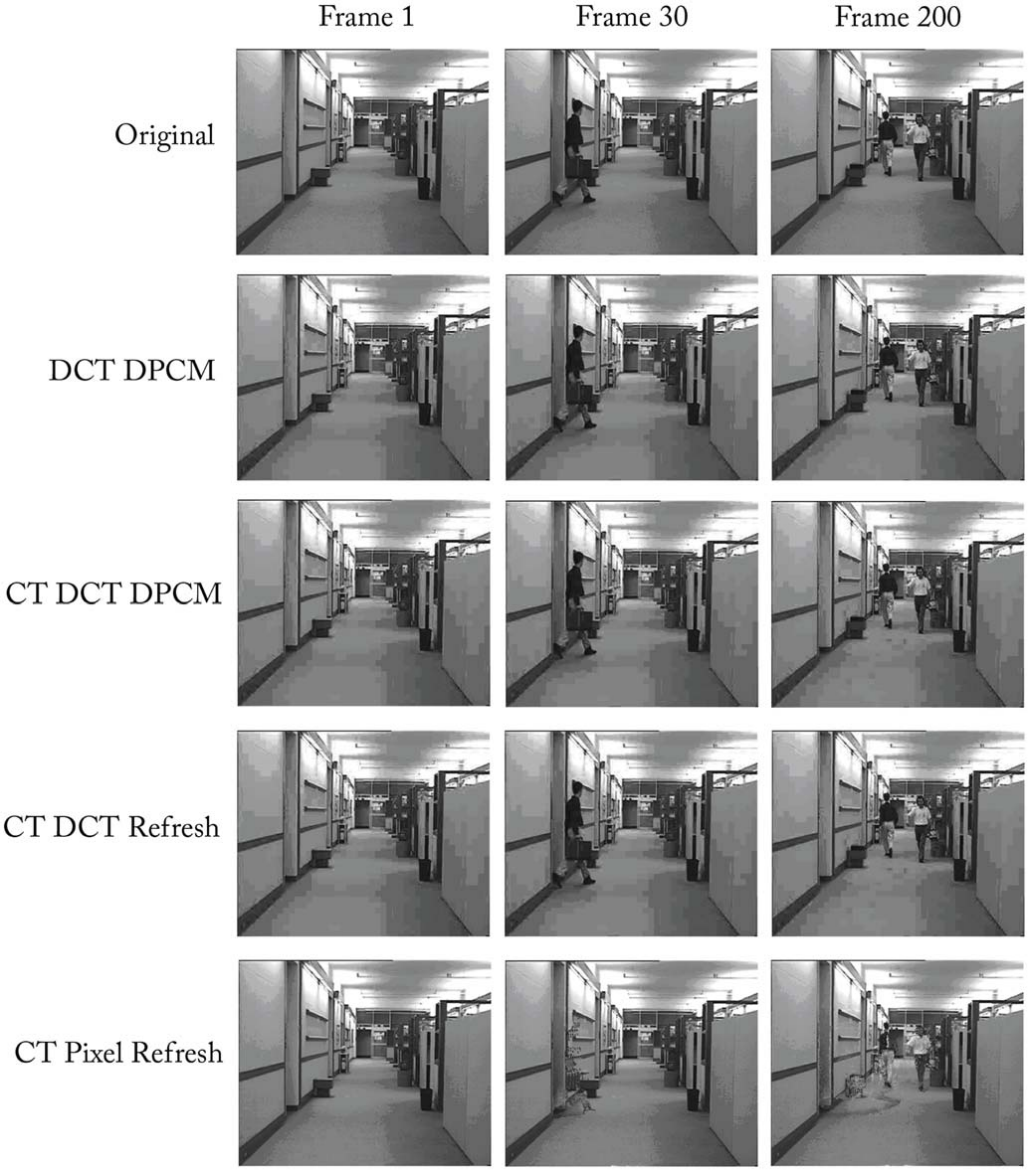
Sample frames from the original video test sequence and the compressed output from each encoder using the parameter *Q = 48*, δ = 5 and Θ = 8 for the DCT encoders and δ = 10 for the CT Pixel Refresh. Frame 1 is the start of the sequence, Frame 30 is where a man begins to enter the scene and Frame 200 is the final image in the video. All of the DCT encoders have similar compression artifacts, mainly a result of heavy DCT quantization. The CT Pixel Refresh coder does not have blocking artifacts, but missing and trailing pixels.

Finally sample outputs of each encoder are shown in Figure 10 for the first frame, the 20^th^ frame and the final 200^th^ frame of the sequence to visually illustrate the compression related distortion. As expected, each of the DCT based encoders look very similar with the typical blocking artifacts from DCT coefficient quantization. The CT Pixel Encoder was the only one to suffer from artifacts from using the focal-plane change detection. Incomplete change detection manifests itself as missing and trailing pixels necessitating the use of a periodic frame fresh to obtain a clear image and reduce error accumulation. As mentioned previously, the use of CT over a whole block largely mitigates this issue since change detection over multiple pixels is much more effective at preserving significant updates while rejecting noise. Consequently, the visual impact of using CT DCT encoding was minimal, compared to the fully closed loop DCT DPCM encoder.

Using the energy consumption figures from parameters extracted from measurements on the system of Figure 2, a simple 128×128 pixel video sensor operating at 10 fps will consume on the order of only 20 mW including RF transmission of the compressed data stream. Future systems that optimize the design of the sensor, perhaps by integrating portions of the spatial transform onto the focal plane [5]–[9], will further reduce this number. The techniques presented here provide the framework for building highly power efficient video sensor systems suitable for battery powered, wireless operation.
